# Network analysis of acute stress reaction in a sample of Chinese male military college students

**DOI:** 10.3389/fpsyt.2023.1082549

**Published:** 2023-08-08

**Authors:** Yue Gong, Zhihua Guo, Hongliang Lu, Xinlu Wang, Yajuan Zhang, Lei Ren, Xia Zhu

**Affiliations:** ^1^School of Psychology, Shaanxi Normal University, Xi’an, China; ^2^Department of Military Medical Psychology, Air Force Medical University, Xi’an, China; ^3^Military Psychology Section, Logistics University of PAP, Tianjin, China; ^4^Military Mental Health Services and Research Center, Tianjin, China

**Keywords:** acute stress reaction, network analysis, expected influence, military college students, central nodes

## Abstract

**Background:**

Acute stress reaction (ASR) following a stressful event is associated with stress-related mental disorders. However, no studies have investigated the relationships between ASR symptom clusters. The present study aimed to provide a fine-grained understanding of the complex relationships among symptom clusters and identify the central symptom clusters of ASR using network analysis.

**Methods:**

The Acute Stress Reaction Scale (ASRS) was used to investigate the network structure of ASR in 1792 Chinese male military college students who were about to participate in an important physical fitness test. We calculated the weights of the edges connecting different symptom clusters and the central indices of 25 symptom clusters in the final network.

**Results:**

There were five strongest edges with significantly higher weights than most other edge weights, including the edges between “Less communication” and “Isolated from others.” The symptom clusters of “Somatic symptoms,” “Hypoprosexia,” and “Anxiety” were found to be the central nodes with the highest expected influences (primary centrality index).

**Conclusion:**

The present study explored the network structure of ASR, revealed complex connections between symptom clusters, and identified central clusters. These findings have important clinical implications, and it is suggested that the three central symptom clusters may be potential targets for effective interventions for ASR.

## 1. Introduction

Acute stress reaction (ASR) comprises a series of mixed, changing, and early physiological and psychological responses after exposure to stressful events or potentially traumatic events (PTEs), mainly presenting with cognitive, emotional, and behavioral changes, as well as somatic symptoms (1–3). For example, symptoms of ASR may include being in a daze, having decreased attention and difficulty concentrating, feeling that objects are unreal, being easily startled, and having irritability, sweating, or heart palpitations (3, 4). These symptoms tend to subside without deliberate intervention in most cases within days. However, in some cases, the symptoms do not remit within days after onset and are followed by mental disorders (3, 4). In the 11th revision of the International Classification of Diseases and Related Health Problems (ICD-11), ASR is conceptualized as a non-disordered response but may be the reason for mental disorders, which still may require intervention (3, 5). An epidemiological study reported that over 70% of individuals might experience a traumatic event globally (6). The incidence of ASR was 44.1% in hospital visitors during the COVID-19 outbreak (7); the incidence was 66.13% in another study (8). ASR was also detected in 42% of help-seekers after an earthquake (9) and 17.2% of soldiers who have deployed to combat (10). Hence, ASR is prevalent. ASR can pose growing public health problems if the symptoms persist for a prolonged duration. For example, recent studies have indicated that the outbreak of COVID-19 caused ASR with varying degrees of severity among medical staff and patients (7, 11, 12). Previous studies have shown that ASR symptoms may become persistent and connected to other stress-related disorders, such as adjustment disorder and post-traumatic stress disorder (PTSD) (3, 13). For instance, one study reported that people were more susceptible to developing early PTSD if diagnosed with ASR after an earthquake (9).

Furthermore, the ASR is of substantial interest from a military perspective because it can cause serious nonbattle attrition (14). While ASR symptoms gradually disappear in most cases, the time and available fighting force are valuable in military situations such as combat. Therefore, the timely and effective resolution of ASR symptoms is critical to military personnel. The ASR in military situations has been studied. With the different natures of important military tasks and exposure factors, the characteristics of ASR in military personnel significantly varied and had a significant impact on the effectiveness when a military task was performed (15–17). Associations between other psychological constructs and ASR in military personnel have been explored, such as the multiple mediation effects of social support and resilience on the relationship between cognitive emotion regulation and ASR in Chinese soldiers (18). Previous studies have also focused on the potential pathophysiological mechanisms underpinning ASR and the etiology of mental disorders related to ASR, such as osteocalcin-mediated ASR by inhibiting parasympathetic tone (19), the engagement of the hypothalamic–pituitary–adrenal axis (20), and ASR as a risk factor for completed suicide (4). However, little is known about ASR itself. In fact, a thorough exploration of the psychopathological processes underpinning ASR (i.e., examining the interactions between symptoms of ASR), which was revealed for the first time in the present study, is essential for developing effective interventions for ASR and preventing related conditions.

Network analysis is a new data-driven method that posits that mental disorders originate from the interactions between symptoms (21–24). Accordingly, symptoms play an active role in triggering and maintaining the corresponding mental disorder as opposed to passively reflecting the condition (25, 26). For example, the Acute Stress Reaction Scale (ASRS)—developed to timely assess ASR in Chinese subjects—includes many symptoms such as nightmares, disorientation, hypoprosexia, anxiety, and somatic or psychiatric symptoms (2). The network perspective on psychopathology understands ASR as a network composed of these interacting symptoms, similar to other publications which have investigated mental disorders, such as PTSD (27), compulsive sexual behavior disorder (28), and problematic smartphone use (29), as networks of interacting symptoms. This approach addresses the problems of previous studies that have commonly measured ASR using simple summing scores from a single-ensemble perspective, an approach that ignores potential interactions between individual symptoms and masks the heterogeneity and the degree of importance of specific symptoms (30–32). Network analysis involves visualizing the relationships between symptoms in the form of a network and helps identify important characteristic symptoms of mental disorders and has been widely used in the field of psychopathology (21, 25, 33, 34). In the visualized network structure, edges represent the partial correlations between symptoms, and nodes represent symptoms of the mental disorder (22, 24, 25). Compared with traditional correlational analysis, network analysis can provide a predictability index for each symptom to determine the controllability of the node and network (35). Controllability of a symptom indicates whether intervention in that symptom through the symptom network is promising (36). When the predictability is high, we can control the symptom via its neighboring symptoms in the network; when the predictability is low, we can directly intervene in it or look for other variables out of the network to control it (35, 37). It also provides centrality indices that identify the central symptoms that play important roles in impacting other symptoms or the entire symptom network, advancing understanding of which symptoms are critical for developing and maintaining mental disorders (25, 38). In other words, it permits the identification of central nodes that significantly impact the whole network and represent potential targets for treatment (38–40).

To date, no study has used network analysis to explore the structure of ASR, and little is known about the internal interactions of ASR symptoms. To address this gap, the present study was conducted to examine the network structure of ASR using network analysis in a military personnel sample. Previous studies have suggested clear gender differences in the ASR (41, 42). Additionally, the majority of military personnel are male. Taken these considerations together, this study chose to focus on males. This study was data-driven without *a priori* hypothesis of relationships among symptom clusters. We aimed to investigate the complex relationships between different symptom clusters at a fine-grained level to advance understanding of the potential pathways between ASR symptom clusters. A second aim was to identify the central symptoms within the ASR network that may be potential targets for effective interventions for ASR. As this study is the first to use network analysis to investigate the network structure of ASR, our work is mainly exploratory.

## 2. Methods

### 2.1. Ethical approval

The study was reviewed and approved by the Medical Ethics Committee of Tangdu Hospital. All participants provided written informed consent prior to participating in the study.

### 2.2. Participants

We chose military college students who were about to participate in an annual military physical fitness test to participate in the study. The test was very important as it was designed to evaluate the servicemen’s physical abilities and was included in their academic performance, which had veto power and was closely related to whether they could graduate smoothly. Since there are gender differences in ASR (41) and the majority of the military personnel are males, we focused on male participants. The ASR was evaluated after the participants were informed that they would undergo the military physical fitness test the next day. According to previous studies, the examination was a real-world stressful event (e.g., final examination) (43, 44), and researchers found that students experienced stress on the day before the final examination (44). Therefore, the likelihood that the participants included in this study would experience acute stress was very high in the face of the important military physical fitness test. All the participants reported that they fully understood the necessity of answering each item honestly and completing the scale independently. A total of 1,910 military college students were recruited from the Air Force Medical University based on the cluster sampling method. The inclusion criteria were as follows: (1) age ≥ 18; (2) gender: male; and (3) provision of informed consent. The exclusion criterion was a history of organic brain damage or mental disorders. A total of 118 female participants were excluded. Thanks to the participants’ excellent obedience, all the scales included were considered valid. The final sample consisted of 1792 male military college students. The average age of participants was 20.88 ± 1.80 years (mean ± SD, range = 18–25 years). The grade ranged from the first year to the fifth grade of the university.

### 2.3. Measures

The Acute Stress Reaction Scale (ASRS) was used to evaluate the severity of ASR. The scale was initially developed and validated in research on individuals participating in earthquake rescues and intensive military training (2, 45). The ASRS was a reliable and appropriate tool for assessing ASR in this study compared to other measures because it was developed using the Chinese language and exhibited satisfactory indices of validity and reliability in the Chinese population, particularly in military personnel (2). The scale comprises six dimensions and 25 symptom clusters, including cognitive changes (nightmares, memory loss, disorientation, indecision, uncertainty, and hypoprosexia), emotional changes (grief, frustration, anger, anxiety, despair, apathy, guilt, helplessness, and depression), behavioral changes (less hygiene, less communication, panic attacks, obsessive behaviors, changes of sleep behaviors, changes in eating habits, and isolated from others), physiological responses (somatic symptoms), psychiatric manifestations (psychiatric symptoms), and work changes (reduced work efficiency). The original internal consistency coefficients for the six dimensions were 0.89, 0.89, 0.84, 0.88, 0.74, 0.79, and 0.85, respectively, and the ASRS had favorable validity (2). The ASRS consists of 112 items, and the participants were asked to answer each item with a “Yes” or “No” response. The ASRS was translated into English and is presented in Supplementary material 1. The internal consistency calculated by 25 symptom clusters of this scale in our sample was excellent (Cronbach’s *α* = 0.94).

### 2.4. Analytical procedures

Descriptive statistics and Cronbach’s alpha coefficient were calculated using SPSS 26, while network analysis was conducted using RStudio (version 1.1.463). Analytic code was available in the attached R scripts.

The R-package *qgraph* was used to construct and visualize the network (46). The network structure was estimated via a Gaussian graphical model (GGM) (47), wherein the edges represent the partial correlation between two nodes after controlling for statistical interference from the remaining nodes. The GGM was estimated based on the non-parametric Spearman rho correlation matrices recommended by a previous study (48). The regularization of the GGM was performed via the graphical least absolute shrinkage and selection operator (LASSO) algorithm (49). This process helps obtain a more stable, sparse, and easy-to-interpret network by shrinking all edges, and edges with trivial partial correlations are identified as spurious and are shrunk to zero (48, 49). Moreover, the tuning parameter of the Extended Bayesian Information Criterion (EBIC) was set to 0.5 to balance the sensitivity and specificity of the extracted actual edges (48, 50). The network was visualized via the Fruchterman-Reingold algorithm (51).

Central nodes within a specific network were used to determine the etiology and intervention targets of specific mental health issues due to their strong connectivity (21). To determine the central nodes, we calculated each node’s expected influence using R-package *qgraph* (46), defined as the sum of weights of all edges linked to a specific node. The higher the expected influence, the more important and influential the node is in the network. Compared with traditional centrality measures such as strength, closeness, and betweenness, the expected influence is more appropriate for determining central nodes in a network with both positive and negative edges (52). Additionally, the betweenness and closeness centrality metrics seem less relevant to psychopathological networks than social networks (23, 53). Moreover, a recent study indicated that only the expected influence successfully predicted how strongly changes in nodes were relevant to changes in the remainder of the nodes compared to other centrality indices (54). Although the expected influence has certain advantages, we still calculated other central indices (i.e., strength, betweenness, and closeness) using R-package *qgraph* (46) to supplement the comprehensive understanding. Importantly, in this study, we used the expected influence index as the primary criterion for determining central nodes. Finally, the node’s predictability was computed using the R-package *mgm* (35). Predictability refers to the degree to which the variance of a node can be explained by all of its neighboring nodes and reflects the controllability of the node because high node predictability indicates that it can be controlled via its neighboring nodes (35, 36).

The robustness of the network was evaluated using the R-package *bootnet* (55). The accuracy of edge weights was examined by computing the 95% confidence intervals (CIs) using a non-parametric bootstrap method (2,000 bootstrap samples), with narrower 95% CIs indicating a more reliable network (56). The stability of centrality indices (i.e., the expected influence, strength, betweenness, and closeness) was assessed by calculating the correlation stability (CS) coefficient using a case-dropping bootstrap approach (2,000 bootstrap samples). The value of the CS coefficient should not be less than 0.25 and preferably higher than 0.5 (55). Furthermore, bootstrapped difference tests (2,000 bootstrap samples) were calculated for edge weights and the node’s expected influence.

## 3. Results

Table 1 presents the mean score, standard deviation, predictability, and expected influence (raw value) for each ASRS symptom cluster.

**Table 1:** Mean score, standard deviation, predictability, and expected influence (raw value) for each symptom cluster of the ASRS.

Symptom cluster	M	SD	Predictability	Expected influence
Nightmares	0.22	0.27	0.44	0.91
Memory loss	0.33	0.33	0.49	0.92
Disorientation	0.14	0.25	0.35	0.65
Indecision	0.35	0.36	0.42	0.78
Uncertainty	0.25	0.29	0.64	1.20
Hypoprosexia	0.39	0.32	0.63	1.31
Grief	0.20	0.31	0.42	0.82
Frustration	0.32	0.30	0.54	1.00
Anger	0.24	0.30	0.46	0.85
Anxiety	0.30	0.34	0.65	1.24
Despair	0.14	0.24	0.54	0.92
Apathy	0.19	0.28	0.45	0.91
Guilt	0.26	0.28	0.37	0.68
Helplessness	0.20	0.28	0.57	1.11
Depression	0.23	0.27	0.61	1.17
Less hygiene	0.11	0.31	0.12	0.37
Less communication	0.15	0.25	0.50	0.87
Panic attacks	0.07	0.15	0.47	0.78
Obsessive behaviors	0.23	0.27	0.43	0.87
Changes of sleep behaviors	0.24	0.23	0.39	0.67
Changes in eating habits	0.13	0.22	0.22	0.50
Isolated from others	0.16	0.27	0.46	0.79
Somatic symptoms	0.16	0.17	0.70	1.33
Psychiatric symptoms	0.06	0.13	0.29	0.51
Reduced work efficiency	0.31	0.40	0.46	0.78

M, mean score; SD, standard deviation.

**Figure 1:**
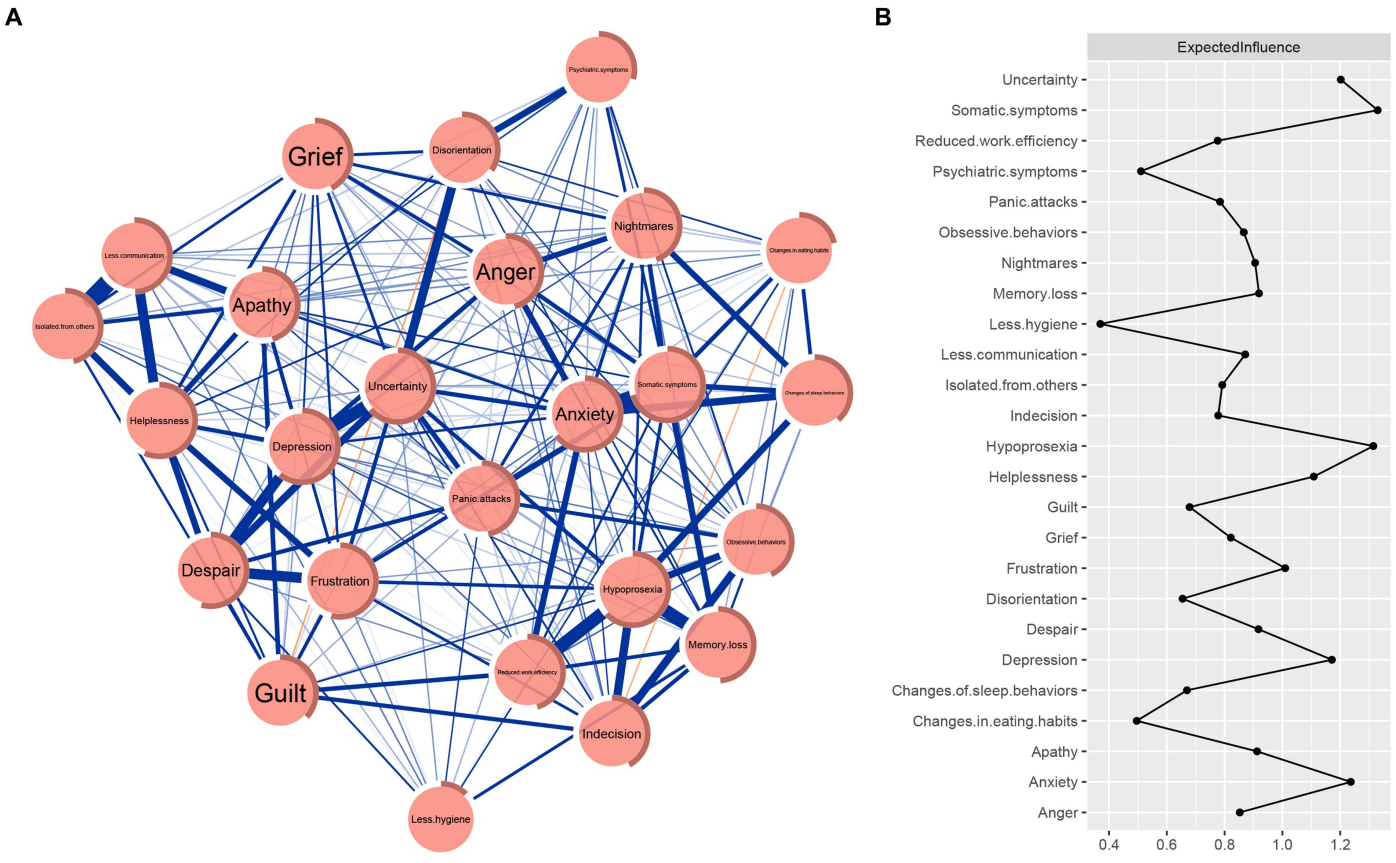
ASR network and raw value of the expected influence for each node. **(A)** ASR network. Blue edges represent positive correlations, and red edges represent negative correlations. The thickness of the edge indicates the strength of the correlation. The ring around each node depicts its predictability. **(B)** Centrality plot depicting the expected influence of each symptom in the network (raw value).

The ASR network is shown in Figure 1A, with several apparent characteristics. First, 207 (69%) edges were non-zero among 300 possible edges, and most of them were positive (only three edges were negative: those between “Guilt” and “Disorientation,” between “Changes in eating habits” and “Indecision,” and between “Anger” and “Despair”), indicating that there were extensive associations between nodes. Second, the five strongest edges existed between “Less communication” and “Isolated from others” (weight = 0.29), between “Anxiety” and “Somatic symptoms” (weight = 0.23), between “Memory loss” and “Hypoprosexia” (weight = 0.22), between “Hypoprosexia” and “Reduced work efficiency” (weight = 0.22), and between “Uncertainty” and “Depression” (weight = 0.21). All edge weights can be seen in Supplementary material 2. Bootstrapped 95% CIs indicated that the estimation of edge weights was accurate and reliable (see Supplementary Figure S1). The bootstrapped difference tests for edge weights showed that the weights of the five strongest edges were significantly higher than the majority of the other edge weights (see Supplementary Figure S2). Third, node predictability was visualized as a ring around each node, with values ranging from 12 to 70%. The nodes “Somatic symptoms,” “Anxiety,” and “Uncertainty” had the highest node predictability (0.70, 0.65, and 0.64, respectively). The average node predictability was 46%, indicating that, on average, 46% of the variance of the nodes in the network could be explained by their neighboring nodes (see Table 1).

The raw values of expected influence for nodes were calculated to assess their centrality and importance in the network (see Table 1 and Figure 1B). The three nodes with the highest expected influences were “Somatic symptoms,” “Hypoprosexia,” and “Anxiety,” indicating that they were the central nodes and were the most important and influential in the network. The CS coefficient of the node’s expected influence was 0.75, indicating that the estimations of the node’s expected influences were adequately stable (see Supplementary Figure S3). Moreover, the results of the bootstrapped difference test for the node’s expected influences showed that the node’s expected influences of “Somatic symptoms,” “Hypoprosexia,” and “Anxiety” were significantly higher than approximately 83.3–91.7% of the other node’s expected influences (see Supplementary Figure S4). The results of other centrality indices (i.e., strength, betweenness, and closeness) for nodes and the corresponding stability of these centrality indices can be found in Supplementary Figures S5, S6. The CS coefficients of the node’s strength, betweenness, and closeness were 0.75, 0.52, and 0.75, respectively. These centrality indices similarly indicated that “Somatic symptoms,” “Hypoprosexia,” and “Anxiety” were the central nodes.

## 4. Discussion

To our knowledge, this is the first study to investigate ASR and visualize complex connections between symptom clusters using network analysis. ASR is defined as a range of psychological and physiological responses to a stressful event and can be considered a predictable indicator of stress-related disorders. It is also closely related to PTSD (2, 57). The present study examined the network structure of ASR to provide a fine-grained understanding of the relationships among its symptom clusters and provides insights into potential targets for effective interventions and treatments of ASR.

According to the results, there were five strongest edges in the network. The strongest edge was between “Less communication” and “Isolated from others,” which is within the behavioral changes dimension. This finding is consistent with a previous study that showed the strongest edge in the network was within each PTSD dimension (e.g., there was a strong edge between “avoidance of thoughts” and “avoidance of reminders” within the avoidance and numbing dimension) (27). The results also revealed a close association between reduced communication and being isolated from others, consistent with the findings of previous studies indicating that communication plays an important role in alleviating social isolation (58, 59). The current study also showed that the second strongest edge was between “Anxiety” and “Somatic symptoms.” This was expected as previous studies have also demonstrated that anxiety is closely related to somatic symptoms (60, 61). One of the strongest edges existed between “Hypoprosexia” and “Reduced work efficiency,” suggesting that decreased attention is related to reduced work efficiency. This finding is concordant with a previous study indicating that reduced work efficiency was positively correlated with hypoprosexia when soldiers engaged in major stressful military tasks (62). In addition, our study showed a strong connection between “Memory loss” and “Hypoprosexia,” manifesting deficits in attention and memory under an acutely stressful condition, consistent with previous studies, indicating that individuals exposed to an acute stressor had impaired attention and memory processes (63, 64). Moreover, there was a strong edge between “Uncertainty” and “Depression,” consistent with previous findings that the feeling of uncertainty is an important psychopathological construct related to depression (65, 66). The average predictability across the whole network was 46%, which is relatively high, implying that the current network was relatively self-determined (35, 67).

This study demonstrated that the central ASR nodes were “Somatic symptoms,” “Hypoprosexia,” and “Anxiety,” partly consistent with a previous study reporting that anxiety is a core symptom of ASR (68). The close relationship between anxiety and somatic symptoms may partially account for the high centrality of somatic symptoms (61, 69). However, the central node “Hypoprosexia” in this study has not been reported previously and is largely exploratory. One conceivable reason is the fact that few studies have focused on ASR, let alone using network analysis. Therefore, further research is necessary to validate these findings. Somewhat surprisingly, our findings do not coincide with previous network analysis studies on PTSD, which have shown emotional reactivity to be the most central node (27, 70). This may be because ASR occurs shortly after a stressful or traumatic event as physiological responses, cognitive changes, and emotional changes simultaneously. In most cases, these ASRs subside over time. However, in some cases, the early responses do not remit, and these individuals are susceptible to prolonged mental disorders, such as PTSD (1, 2). Thus, while ASR and PTSD seem similar, they are radically different, and it is not surprising that the central symptoms of the two conditions are different. Altogether, the present study provides novel insights into potential targets for interventions and treatments of ASR.

These findings will be of significance in the theory and clinic. Regarding theoretical implications, investigating the fine-grained relationships among the individual symptom clusters of ASR can provide preliminary insights into the complex interactions between strongly connected symptom clusters. The connections between pairs of symptom clusters advance our understanding of the psychopathological pathways between symptoms of ASR and partly elucidate the mechanisms underlying the development and maintenance of ASR. Regarding clinical implications, it has been reported that central symptoms in the network may play an important role in activating other symptoms and strongly influence the development and maintenance of mental disorders (25, 52). Nodes central to the network are promising targets for intervention and treatment insofar as they can potentially interrupt the overall network (25, 37, 38, 71, 72). Consequently, this finding indicates that interventions targeting “Somatic symptoms,” “Hypoprosexia,” and “Anxiety” may effectively alleviate and treat ASR symptoms. In addition, the predictability of “Somatic symptoms” and “Anxiety” was high, suggesting that these two nodes can be largely controlled by their respective neighboring nodes (35, 36, 67). This indicates that we could not only intervene in “Somatic symptoms” and “Anxiety” directly but also via their strong connecting nodes in the network, such as targeting “Changes of sleep behaviors” and “Anger” for controlling “Anxiety” (see Figure 1A and Supplementary material 2).

Although the present study helps understand the relationships between symptom clusters of ASR at a fine-grained level, some limitations must be acknowledged. First, the study was based on cross-sectional data, precluding any claims about causality. Future studies should use longitudinal experimental designs to examine the causality of these symptoms. Second, the network model in this study was data-driven and exploratory, and a general problem with any exploratory model is the generalizability of the network to other samples. The findings of this study were based on Chinese male military college students. Therefore, it is not known how generalizable our results are to other populations. Future studies should determine the replicability of the results. The third limitation is that some students may not have become particularly stressed before the test. Future studies could strengthen the methodology by including objective measures of stress (e.g., blood pressure and heart rate) to confirm stress levels. Fourth, the ASRS we used was a self-report scale, which might be susceptible to subjective response biases and social approval effects (26, 73). Thus, our findings may differ from the symptoms following an actual traumatic or PTE and must be interpreted cautiously. Fifth, the network structure of ASR in the current study is specific to the scale we used. The ASRS cannot contain all aspects of the ASR, and different scales may produce a different network structure. Sixth, because this study was a cross-sectional survey focusing on ASR, there is few information on how investigated ASR profile can influence/predict future occurrence of mental health problems or their absence. This is also one of the directions for our future longitudinal research. Finally, like the first limitation mentioned above, although we have identified potential targets for treating ASR, it remains to be seen whether interventions targeting these symptom clusters will be effective and warrants additional research.

## 5. Conclusion

The present study is the first to investigate the network structure of ASR in Chinese male military college students using network analysis. We found many connections between symptom clusters, and there were five strongest edges, providing a fine-grained understanding of the complex relationships within ASR. The symptom clusters “Somatic symptoms,” “Hypoprosexia,” and “UncertaintyAnxiety” were determined to be the central symptoms, which may exert a more significant influence on the development and maintenance of ASR than other symptom clusters. Therefore, treatments of ASR that target these central symptom clusters may be the most effective in alleviating ASR and maximizing the impact of an intervention.

## Data availability statement

The raw data supporting the conclusions of this article will be made available by the authors, without undue reservation.

## Ethics statement

The studies involving human participants were reviewed and approved by the Medical Ethics Committee of Tangdu Hospital—an Affiliated Hospital of Air force Medical University. The patients/participants provided their written informed consent to participate in this study.

## Author contributions

YG, ZG, LR, and XZ conceived the study. YG and XZ performed the data collection. HL, XW, YZ, and LR performed data analysis. YG and ZG wrote the draft of the manuscript. LR and XZ obtained funding and contributed to the manuscript revision. All authors contributed to the article and approved the submitted version.

## Funding

This work was supported by the Major Project of Medicine Science and Technology of PLA (AWS17J012).

## Conflict of interest

The authors declare that the research was conducted in the absence of any commercial or financial relationships that could be construed as a potential conflict of interest.

## Publisher’s note

All claims expressed in this article are solely those of the authors and do not necessarily represent those of their affiliated organizations, or those of the publisher, the editors and the reviewers. Any product that may be evaluated in this article, or claim that may be made by its manufacturer, is not guaranteed or endorsed by the publisher.
