# Supplementary material for: Network analysis of acute stress reaction in a sample of Chinese male military college students
Source: Front Psychiatry. 2023 Aug 8;14:1082549. doi: 10.3389/fpsyt.2023.1082549 (PMC10444979; doi:10.3389/fpsyt.2023.1082549)
Supplement: Supplementary file 1 [file Data_Sheet_1.DOCX]

**Please answer each item with a “Yes” or “No” response.**

1 Do you feel confused and don't know what to do?

2 Do you feel confused in familiar places and don't know where to go?

3 Do you feel that you can't control yourself when doing one thing and don't think about other things?

4 Do you feel miserable because it is difficult to make a decision?

5 Do you feel that you have a harder time remembering things?

6 Do you often argue with others over small things?

7 Do you feel that you are depressed and don't want to do anything?

8 Do you feel that you can't do anything?

9 Do you feel that no one can help you?

10 Are you always afraid to compete with others?

11 Do you have no appetite?

12 Do you have trouble falling asleep and lying down for a long time?

13 Are you unwilling to wash your face or shower?

14 Are you unwilling to be with others?

15 Do you now talk less?

16 Do you now feel restless?

17 Do you now feel your heart beating faster?

18 Do you now feel chills or fever?

19 Do you now have unnecessary thoughts or words swirling in your head?

20 Do you feel lost and not know the meaning of life?

21 Do you feel that familiar places suddenly feel strange?

22 Do you feel that your understanding is not as good as before?

23 Do you often forget where you put your things?

24 Do you often have strange dreams?

25 Do you feel that you are now emotional and uncontrollable?

26 Do you often think about death?

27 Do you feel hopeless about the future?

28 Do you feel that no one cares about you?

29 Do you feel that you have no courage to face the future?

30 Do you often feel nauseous or have an upset stomach?

31 Do you wake up earlier than usual, which affects your mental state during the day?

32 Do you feel reluctant to talk to others or feel bored when talking to others?

33 Do you feel bouts of fear?

34 Do you feel chest tightness and shortness of breath?

35 Do you feel heavy now?

36 Do you have to do things slowly to make sure they are done correctly?

37 Do you feel restless?

38 Do your mind often wander?

39 Do you often hesitate to do things?

40 Do you often feel like you forget the things you just finished?

41 Do you often get confused between dreams and reality and feel afraid?

42 Do you get angry more easily than ever?

43 Do you feel miserable and always want to cry?

44 Do you feel like your life is empty and you feel worthless?

45 Do you feel like you can't succeed no matter how hard you try?

46 Do you feel that there is no one you can trust?

47 Do you have to give in to external forces?

48 Do you now have trouble sleeping all night?

49 Do you now always want to be alone?

50 Do you now people talk to you and you don't talk to them?

51 Are you now always afraid to be alone and afraid to turn off the light to sleep?

52 Do you feel like your world is spinning, or suddenly faint?

53 Do you feel a lump in your throat now?

54 Do you have to double-check everything?

55 Do you feel confused?

56 Do you feel that you suddenly come to a strange place and don't know where you are?

57 Do you often pass by familiar people and only realize it later?

58 Do you feel more at ease when others help you make decisions?

59 Do you feel like there are periods of time when your experience is completely blank?

60 Do you often wake up feeling weak and depressed?

61 Do you get into fights more often than ever?

62 Do you feel like you can't cry anymore?

63 Do you think you should be punished?

64 Do you feel that you have no meaning to live?

65 Do you feel that you are always alone?

66 Do you feel that you have no resistance to the situation?

67 Do you throw up when you eat?

68 Do you sleep less and wake up easily?

69 Do you want to get away from others by giving up work?

70 Do you prefer to be mute?

71 Do you fall faint because of excessive fear?

72 Do you feel dizzy and swollen with blood?

73 Do you have diarrhea or constipation?

74 Do you now have to wash your hands repeatedly and close the door repeatedly, etc.?

75 Do you have the urge to hit or hurt others?

76 Do you feel lonely when you are with others?

77 Do you have the urge to break or destroy things?

78 Do you feel like your face is tight all the time?

79 Do you feel physically sick now, but don't know which part is it?

80 Do you still feel tired after you wake up?

81 Do you feel that your attention range is smaller and you can't pay attention to details of things?

82 Do you feel distracted and unable to concentrate?

83 Do you have increased frequency of urination and urgency?

84 Do you have incontinence caused by tension?

85 Do you have stomach pain?

86 Do you keep nervous and unable to relax?

87 Do you think you might go crazy?

88 Do you think you are useless?

89 Do you hear voices but no one else can hear?

90 Do you feel that someone is controlling your thoughts?

91 Do you shake with nervousness and cannot control?

92 Do you move around restlessly?

93 Do you always think about the present situation and you can't help it?

94 Do you feel weak or tired easily?

95 Do you feel that you don't care about others?

96 Do you see things but no one else can?

97 Do your feel so nervous that hands shake?

98 Do you become more careless than before?

99 Do you feel that your ability to solve problems is less than before?

100 Do you feel that you are not doing your best?

101 Do you feel that everything has nothing to do with you?

102 Do you feel that someone is monitoring you?

103 Do you have a nervous headache or a backache?

104 Do you feel that your usual work skills have decreased?

105 Do you feel that you are more nervous and anxious than usual?

106 Do you feel that you are not playing your role?

107 Do you feel less happy or sad?

108 Do you feel like someone is trying to hurt you?

109 Do you talk faster than usual and you can't help it?

110 Do you feel like you are easily upset or frightened?

111 Do you feel guilty?

112 Are you sure someone knows what you're thinking?
