# Supplementary material for: Network analysis of acute stress reaction in a sample of Chinese male military college students
Source: Front Psychiatry. 2023 Aug 8;14:1082549. doi: 10.3389/fpsyt.2023.1082549 (PMC10444979; doi:10.3389/fpsyt.2023.1082549)
Supplement: Supplementary file 3 [file Data_Sheet_3.docx]

**Supplementary Materials 3**

1. Figure S1. Accuracy of edge weights

2. Figure S2. Bootstrapped difference test for edge weights

3. Figure S3. Stability of node expected influences

4. Figure S4. Bootstrapped difference test for node expected influences

5. Figure S5. Centrality plot depicting the strength, betweenness and closeness of each node within the present network

6. Figure S6. Stability of other node centrality indices (i.e., strength, betweenness and closeness)

Figure 1. Accuracy of edge weights

*Note*: The red line depicts the sample edge weights and the gray bar depicts the bootstrapped confidence interval.

Figure 2. Bootstrapped difference test for edge weights

*Note*: Gray boxes indicate edge weights that do not differ significantly from one another, while black boxes indicate edge weights that do differ significantly. Blue and red boxes on the diagonal correspond to edge weights with positive and negative correlations, respectively.

Figure 3. Stability of node expected influences

*Note*: The red bar represents the average correlation between node expected influences in the full sample and subsample with the red area depicting the 2.5th quantile to the 97.5th quantile.

Figure 4. Bootstrapped difference test for node expected influences

*Note*: Gray boxes indicate node expected influences that do not differ significantly from one another, while black boxes indicate node expected influences that do differ significantly. The number in the white boxes (i.e., diagonal line) represent the value of node expected influences.

Figure 5. Centrality plot depicting the strength, betweenness and closeness of each node within the present network

Figure 6. Stability of other node centrality indices (i.e., strength, betweenness and closeness)

*Note*: The red bar represents the average correlation between node centrality indices in the full sample and subsample with the red area depicting the 2.5th quantile to the 97.5th quantile. The CS coefficient of the node’s strength, betweenness and closeness were 0.75, 0.52 and 0.75, respectively.
